# Anti-photoaging properties of the phosphodiesterase 3 inhibitor cilostazol in ultraviolet B-irradiated hairless mice

**DOI:** 10.1038/srep31169

**Published:** 2016-08-03

**Authors:** Ha Neui Kim, Chan Hee Gil, Yu Ri Kim, Hwa Kyoung Shin, Byung Tae Choi

**Affiliations:** 1 Department of Korean Medical Science, School of Korean Medicine, Pusan National University, Yangsan 50612, Korea; 2 Korean Medical Science Research Center for Healthy-Aging, Pusan National University, Yangsan 50612, Korea; 3 Division of Meridian and Structural Medicine, School of Korean Medicine, Pusan National University, Yangsan 50612, Korea

## Abstract

We investigated whether cilostazol, an activator of cyclic adenosine monophosphate (cAMP)-dependent intracellular signaling, could inhibit ultraviolet B (UVB) irradiation-induced photoaging in HR-1 hairless mice. Cilostazol decreased wrinkle formation and skin thickness in UVB-irradiated mice, as well as increased staining of collagen fibers and inhibition of reactive oxygen species (ROS) formation in the skin. Moreover, the proteolytic activities of gelatinase matrix metalloproteinase (MMP)-9 and collagenase MMP-3 were significantly decreased in UVB-irradiated mice treated with cilostazol. Western blotting showed that UVB-induced activation of p38 mitogen-activated protein kinases (MAPK) and nuclear factor (NF)-κB was significantly inhibited by cilostazol, whereas the activation of Akt was significantly enhanced by cilostazol. Confirmation of localized protein expression in the skin revealed marked p38 MAPK and NF-κB activation that was mainly detected in the dermis. Marked Akt activation was mainly detected in the epidermis. Our results suggest that cilostazol may have anti-photoaging effects on UVB-induced wrinkle formation by maintaining the extracellular matrix density in the dermis, which occurs via regulation of ROS and related p38 MAPK and NF-κB signaling, and subsequent down-regulation of MMPs. Therefore, cilostazol may protect against photoaging-induced wrinkle formation.

Solar radiation comprises >10% ultraviolet rays (UV). UV radiation causes overproduction of reactive oxygen species (ROS) in the skin, which play a key role in oxidative damage to the skin. This is considered the major cause of photoaging^1^,^2^. Wrinkle formation is a striking feature of photoaging and is caused by the degradation of components of the extracellular matrix of the skin, such as collagen fibrils and gelatin fibers^3^,^4^. The generated ROS from UV radiation can cause subsequent alterations in cellular responses, inducing the activation of matrix metalloproteinases (MMPs), which degrade the extracellular matrix^5^,^6^.

Generation of ROS in the skin plays a critical role in the mitogen-activated protein kinases (MAPK) and phosphatidylinositol-3 kinase (PI3K)/Akt-mediated signaling pathways triggered by UV radiation^4^,^7^,^8^,^9^. These kinases stimulate the transcription factors activator protein (AP)-1 and nuclear factor (NF)- κB^10^,^11^, and ultimately participate in the induction of MMP activation^5^,^6^, leading to wrinkle formation in photoaged skin^12^,^13^. Thus, regulation of ROS formation and the signaling cascade related to UV irradiation have profound utility as a treatment for skin photoaging.

Cilostazol is a selective inhibitor of type 3 phosphodiesterase (PDE3), which prevents degradation of intracellular cyclic adenosine monophosphate (cAMP) and produces various pleiotropic effects via the PDE3-cAMP signaling cascade^14^,^15^. Cilostazol is widely approved for the treatment of intermittent claudication in peripheral arterial disease, and provides protective effects as a secondary prevention of ischemic stroke^16^,^17^,^18^. Recently, cilostazol showed prominent inhibition of intracellular ROS production by inhibiting nicotinamide adenine dinucleotide phosphate oxidase activity^13^,^19^,^20^,^21^,^22^.

Blocking the ROS-related signaling activated by UV irradiation could be an additional strategy for preventing photoaging. We therefore hypothesized that cilostazol might have protective properties against skin photoaging by quenching the production of ROS and the related signaling cascade. We have previously observed that cilostazol shows anti-wrinkle effects in an animal model of photoaging. In the present study, we investigated the potential effects of cilostazol on UVB-induced wrinkle formation and the underlying molecular mechanism by histological analysis and assessment of ROS-dependent kinases, loss of collagen fibers, and the subsequent production of MMPs in the skin of hairless mice.

## Results

### The effects of cilostazol on wrinkle formation in UVB-irradiated hairless mice

**Figure 1:**
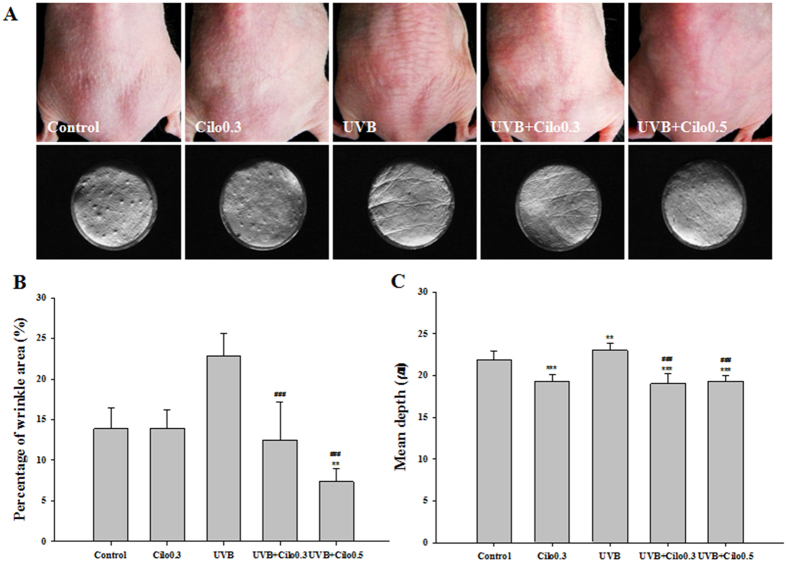
The effect of cilostazol on visible skin condition and wrinkle formation in the dorsal skin of UVB-irradiated mice. (**A**) Photographs and replicas of the mouse dorsal skin. (**B,C**) Histogram of replica analysis. Treatment with cilostazol improved visible skin condition and reduced the percentage of wrinkles per unit area and the mean depth of wrinkles in UVB-irradiated mice. ^**^P < 0.01 and ^***^P < 0.001 vs. control mice; ^###^*P* < 0.001 vs. UVB-irradiated mice.

Skin conditions revealed by photography showed that the dorsal skin of UVB-irradiated mice was rough and flaky compared with that of control mice. However, reduced flakiness and roughness were observed in UVB-irradiated mice treated with cilostazol compared that in mice exposed to UVB irradiation alone (Fig. 1A). Analysis of wrinkle formation in silicon replicas revealed that the wrinkles of control mice were thin and shallow, while those of UV-irradiated mice were thick and deep. Moreover, UVB-irradiated mice showed an increase in the percent area of wrinkles and significant mean depth of wrinkles compared with that observed for the control mice. These changes were attenuated by cilostazol treatment (Fig. 1B,C). Analysis of the silicon replicas suggests that cilostazol may show attenuation of UVB-induced wrinkle formation.

### The effects of cilostazol on skin thickness and collagen fiber density in UVB-irradiated hairless mice

**Figure 2:**
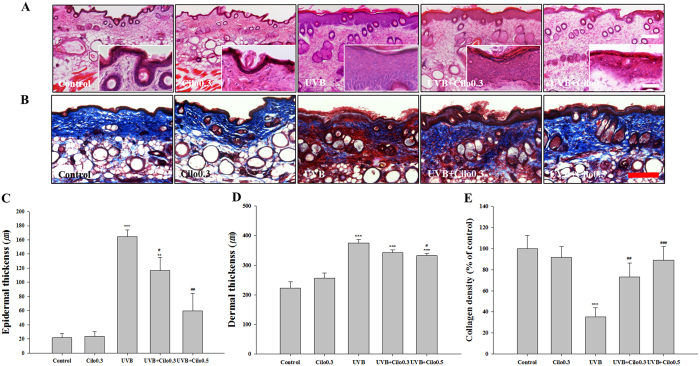
The effect of cilostazol on epidermal and dermal thickness and density of collagen fibers in the dorsal skin of UVB-irradiated mice. (**A**) Hematoxylin and eosin staining and (**B**) Masson’s trichrome staining. Scale bar = 200 μm. Scale bar in rectangular box = 50 μm. (**C,D**) Histogram of hematoxylin and eosin staining. (**E**) Histogram of Masson’s trichrome staining. Treatment with cilostazol significantly suppressed the UVB irradiation-induced increase in epidermal and dermal thickness and prevented UVB-induced loss of collagen fibers. ^**^P < 0.01 and ^***^P < 0.001 vs. control mice; ^#^*P* < 0.05, ^##^*P* < 0.01, and ^###^*P* < 0.001 vs. UVB-irradiated mice.

Histological analysis showed a thicker epidermis and dermis in UVB-irradiated mice compared to that in control mice. UVB-irradiated mice treated with cilostazol showed marked recovery from these pathological changes compared with that in non-treated UVB-irradiated mice (Fig. 2A,C,D). UVB-irradiated mice showed a significant decrease in the abundance and density of collagen fibers in the dermis compared with that in control mice as assessed by Masson’s trichrome staining. However, treatment with cilostazol inhibited the UVB irradiation-induced loss of collagen fibers (Fig. 2B,E). These results show that cilostazol can attenuate UVB irradiation-induced skin thickening and collagen fiber loss, suggestive of a protective effect of cilostazol against UVB-induced skin damage.

### The effects of cilostazol on ROS production in UVB-irradiated hairless mice

**Figure 3:**
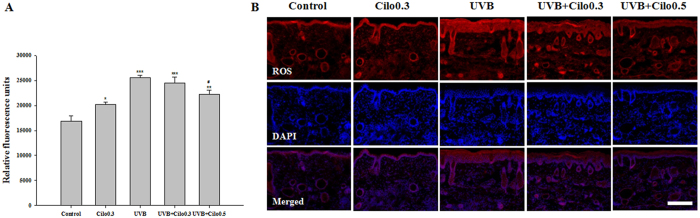
The effect of cilostazol on the production of ROS in the dorsal skin of UVB-irradiated mice. (**A**) Histogram of total ROS assay. ^*^P < 0.05, ^**^P < 0.01, and ^***^P < 0.001 vs. control mice; ^#^*P* < 0.05 vs. UVB-irradiated mice. (**B**) Immunohistochemical staining of ROS. Scale bar = 200 μm. The increased ROS free radical activity was significantly inhibited by cilostazol treatment and was confirmed by fluorescence expression (red) in the dorsal skin.

A significant increase in total ROS production measured in relative fluorescence units (RFU) was observed in UVB-irradiated mice compared to that in control mice. However, 0.5% cilostazol markedly decreased UVB irradiation-induced ROS formation (Fig. 3A). We confirmed the distribution of ROS in the skin and observed that UVB-irradiated mice had significantly increased production of ROS in both the epidermis and the dermis. However, treatment with cilostazol resulted in a significant decrease in UVB irradiation-induced ROS production (Fig. 3B). The significant decrease in ROS in the skin, especially in the epidermis, the extracellular matrix, and cellular components of the dermis, suggests that cilostazol attenuates UVB-induced production of ROS.

### The effects of cilostazol on the proteolytic activity of MMP-9 and MMP-3 in UVB-irradiated hairless mice

**Figure 4:**
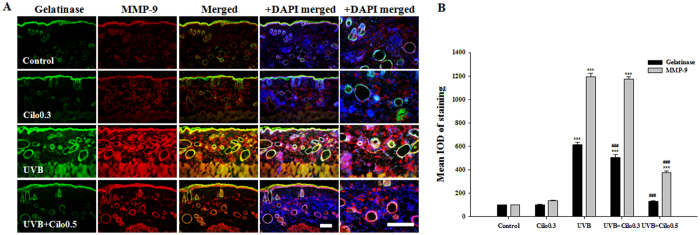
The effect of cilostazol on gelatinase and MMP-9 activity in the dorsal skin of UVB-irradiated mice. (**A**) Images of the *in situ* zymographic analysis of gelatinase and MMP-9, and (**B**) histogram of the IOD measurement. Treatment with cilostazol significantly suppressed gelatinase activity and MMP-9 expression in the dorsal skin of UVB-irradiated mice. ^***^P < 0.001 vs. control mice and ^###^*P* < 0.001 vs. UVB-irradiated mice. Scale bar = 200 μm.

*In situ* zymography showed that gelatinolytic activity co-localized with MMP-9 reactivity. The expression of fluorescent products was mainly observed in keratinocytes of the epidermis and in hair follicles in the control mice. In contrast, the skin of UVB-irradiated mice showed greater fluorescence than the skin of non-irradiated mice, particularly for gelatinolysis in the extracellular matrix of the dermis. However, UVB-irradiated mice treated with cilostazol showed a significant decrease in skin fluorescence intensity related to gelatinolysis compared with that in non-treated UVB-irradiated mice, indicating that cilostazol inhibited the proteolytic activity of MMP-9 (Fig. 4). Collagenolytic activity also co-localized with MMP-3 activity, and the expression fluorescence related to the MMP-3 activity in the control and UVB-irradiated mice was similar to that of the gelatinolytic activity. Mice irradiated with UVB and treated with cilostazol showed decreased collagenolytic activity for MMP-3 (Supplementary Fig. 1). Thus, *in situ* zymography analysis suggests that cilostazol prevents the proteolytic activity of the gelatinase MMP-9 and collagenase MMP-3 due to UVB-induced skin damage.

### The effects of cilostazol on the expression of ROS-related signaling in UVB-irradiated hairless mice

**Figure 5:**
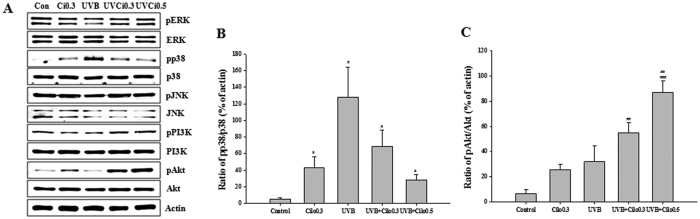
The effect of cilostazol on MAPK and PI3K/Akt expression in the dorsal skin of UVB-irradiated mice. (**A**) Western blotting and (**B,C**) relative densities of p38 MAPK and Akt. UVB irradiation-induced upregulation of the expression of phosphorylated p38 was significantly decreased by cilostazol treatment, whereas phosphorylated Akt expression was increased by cilostazol treatment. ^*^P < 0.05, ^**^P < 0.01, and ^***^P < 0.001 vs. control mice; ^#^*P* < 0.05 and ^##^*P* < 0.01 vs. UVB-irradiated mice.

**Figure 6:**
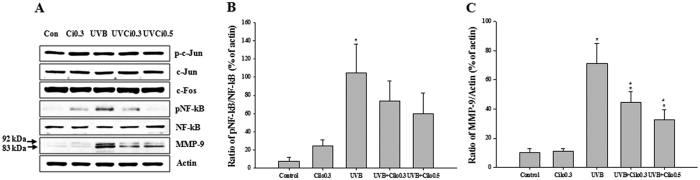
The effect of cilostazol on the expression of the transcription factors AP-1 and NF-κB with MMP-9 in the dorsal skin of UVB-irradiated mice. (**A**) Western blotting and (**B,C**) relative densities of NF-κB and MMP-9. UVB irradiation significantly increased the expression of phosphorylated NF-κB and MMP-9, which was decreased by cilostazol treatment. ^*^P < 0.05 vs. control mice and ^#^*P* < 0.05 vs. UVB-irradiated mice.

Western blot analysis of the expression of ROS-related signaling molecules revealed no significant changes in the expression of total proteins. However, among the MAPK analyzed, in mice exposed to UVB the expression of phosphorylated p38 MAPK was significantly increased. This increase in expression was attenuated by cilostazol treatment. In contrast, a marked increase in phosphorylated Akt expression and a slight increase in phosphorylated PI3K were observed in mice treated with UVB and cilostazol compared to that in UVB-irradiated mice (Fig. 5). Regarding the expression of transcription factors, there was no significant difference in phosphorylated c-Jun between all treatment groups. However, the upregulated expression of phosphorylated NF-κB was slightly decreased in UVB-irradiated mice treated with cilostazol. We found a decreased expression of MMP-9 (both the 92 kDa pro-enzyme form and 83 kDa active form) in UVB-irradiated mice treated with cilostazol compared with that in non-treated UVB-irradiated mice (Fig. 6). Western blot analysis indicated that cilostazol had protective activity against UVB-induced skin damage by inhibiting p38 MAPK, Akt, and NF-κB expression.

### The effects of cilostazol on the localization of p38 MAPK, Akt, and NF-κB in UVB-irradiated hairless mice

**Figure 7:**
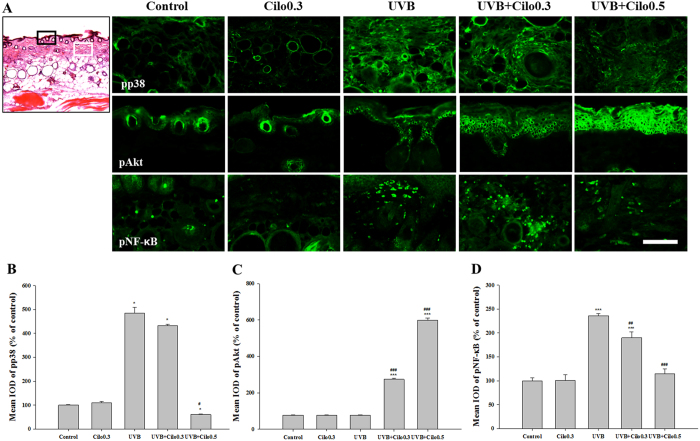
Immunohistochemical analysis of the expression of p38 MAPK, Akt, and NF-κB in the dorsal skin of UVB-irradiated mice. (**A**) Images of immunohistochemically stained skin sections. The expression of phosphorylated Akt was mainly detected in the epidermis (black rectangular box) while phosphorylated p38 MAPK and NF-κB were mainly detected in the dermis (white rectangular box). Scale bar = 100 μm. (**B–D**) Histogram analysis of the IOD measurement of (**B**) phosphorylated p38 MAPK, (**C**) Akt, and (**D**) NF-κB. The significant increase in phosphorylated p38 MAPK and NF-κB by UVB irradiation was attenuated by cilostazol treatment. ^*^P < 0.05 and ^***^P < 0.001 vs. control mice; ^#^*P* < 0.05, ^##^*P* < 0.01, and ^###^*P* < 0.001 vs. UVB-irradiated mice.

Analysis of immunoreactive sites in the skin revealed that phosphorylated Akt was mainly expressed in the epidermis. Furthermore, immunohistochemical analysis showed a significantly increased expression of Akt in UVB-irradiated mice treated with cilostazol. Moreover, expression of phosphorylated p38 and NF-κB was mainly observed in the extracellular matrix and cellular components of the dermis. The expression of phosphorylated p38 and NF-κB was markedly increased in UVB-irradiated mice, which was attenuated by cilostazol treatment (Fig. 7). Because formation of deep wrinkles is mainly associated with the degradation of the extracellular matrix of the dermis, these results indicate that cilostazol treatment exerts protective activity against UVB-induced wrinkle formation, and mainly involves p38 MAPK and transcription factor NF-κB.

### The protective effects of cilostazol against UVB-induced wrinkle formation in UVB-irradiated hairless mice

**Figure 8:**
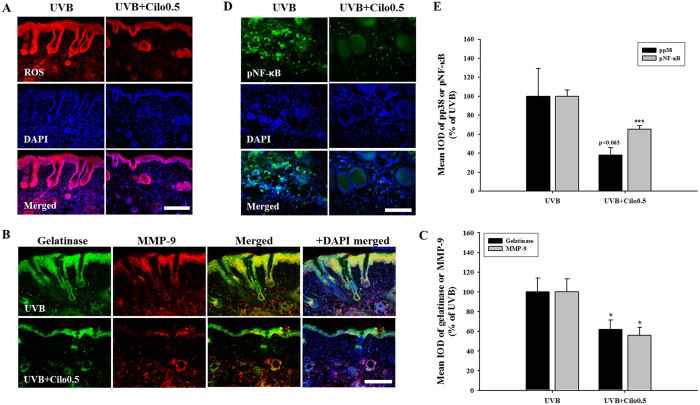
The protective effects of cilostazol against skin damage in UVB-irradiated mice. (**A**) Immunohis-tochemical staining of ROS. Increased ROS activity was inhibited by cilostazol treatment. Scale bar = 200 μm. (**B**) Images of the *in situ* zymographic analysis of gelatinase and MMP-9 and (**C**) the related histogram analysis. Skin treated with cilostazol showed significant suppression of gelatinase activity and MMP-9 expression. ^*^P < 0.05 vs. non-treated skin with cilostazol. Scale bar = 200 μm. (**D**) Immunohistochemical images of NF-κB and (**E**) the related histogram analysis. The significant increase in phosphorylated p38 MAPK and NF-κB by UVB irradiation was attenuated by cilostazol treatment. ^***^P < 0.001 vs. non-treated skin with cilostazol. Scale bar = 100 μm.

To normalize the background signal intensity between control and cilostazol-treated mice, we applied cilostazol lotion on one half of the dorsal skin of a hairless mouse, using the other, non-treated half as control. We performed ROS detection, *in situ* zymography, and pp38 and pNF-kB staining identically in each dorsal skin half. Skin treated with cilostazol showed a significant decrease in ROS production compared to that in the non-treated skin of UVB-irradiated hairless mice. Skin treatment with cilostazol also significantly decreased skin fluorescence intensity related to gelatinolysis. The upregulated expression of phosphorylated p38 MAPK and NF-κB in non-treated skin was significantly decreased by cilostazol treatment (Fig. 8). These results indicate that UVB-induced skin photoaging markers as measured by fluorescent signal quantification was markedly decreased by cilostazol treatment.

## Discussion

Cilostazol is used as an antiplatelet drug for the treatment of intermittent claudication and for the secondary prevention of ischemic stroke^18^. To our knowledge, our work is the first to provide clear *in vivo* evidence that cilostazol treatment is also effective in attenuating UVB irradiation-induced skin photoaging. The mechanism underlying protection against wrinkle formation by cilostazol potentially involves the inhibition of the UVB-induced ROS signaling cascade via cAMP-dependent p38 and NF-κB that leads to MMP-9 expression. We therefore conclude that cilostazol may be a useful therapeutic to prevent UVB-induced skin aging.

UV radiation comprises UVA (320–400 nm) and UVB (280–320 nm) rays that reach the earth’s surface. UVB is the most biologically damaging type of UV radiation^23^,^24^. Longer-wavelength UVA rays generally penetrate to the deep dermal layers of the skin^2^. In contrast, shorter-wavelength UVB rays penetrate the epidermis and are completely absorbed in the upper dermis. Chronic exposure to UVB causes various skin disorders, including photoaging that is characterized by wrinkles and skin laxity^2^,^7^,^25^.

UVB exposure affects the phenotype of the skin structure either by directly affecting the keratinocytes of the epidermis or indirectly by remodeling of the extracellular matrix^3^,^4^,^26^. UVB irradiation increases stratum corneum thickness, collagen fragmentation, and matrix-degrading metalloproteinases in the dermis^4^,^27^,^28^. These morphological changes appear as a loss of elasticity and wrinkling of the skin^29^. Moreover, wrinkle formation due to the degradation of the extracellular matrix is a striking feature of skin photoaging^3^,^4^.

In the current study, treatment with cilostazol showed positive effects on skin photoaging by inhibiting wrinkle formation in UVB-irradiated HR-1 mice. The area percentage and mean depth of the wrinkles were significantly attenuated by cilostazol treatment. Thus, these effects are closely associated with the protective effects of this drug on UVB irradiation-induced wrinkle formation. Cilostazol treatment also showed a protective effect against UVB-induced skin damage by balancing epidermal and dermal thickness and maintaining collagen density. Although quantification of dermis thickness cannot be regarded as direct evidence of the function of cilostazol because of the specific hair cycle stage, the epidermal thickness and the preservation of collagen density suggest a potential involvement of PDE3-cAMP signaling in these protective effects. Therefore, we next investigated the correlative molecular mechanism underlying UVB-induced wrinkle formation and cilostazol-induced cAMP signaling.

UVB penetrates the epidermal layer and causes over-production of ROS, which are considered the main mediators of oxidative damage in photoaging^4^,^30^. ROS play a major role in wrinkle formation in photoaging by initiating and driving the signaling events that lead to induction of MMPs, which degrade the extracellular matrix in response to UV exposure^4^. This imbalance of ROS in the skin is correlated with reduced cAMP-dependent phosphorylation^31^.

Cilostazol increases the intracellular second messenger cAMP and subsequently activates cAMP-dependent signaling^14^. Cilostazol has antioxidant effects and shows prominent inhibition of ROS production^32^,^33^. Application of cilostazol lotion containing components similar to those used in our current study showed that cilostazol was absorbed percutaneously and was retained, even in the inner skin layer, and caused significant changes in cAMP levels in the skin^34^. In the present study, treatment with cilostazol inhibited UVB-induced ROS production in the skin, which was consistent with our hypothesis.

Fragmentation of collagen due to degradation is an important step that contributes to wrinkle formation, and is the result of excessive expression levels and activity of MMPs in response to UV irradiation^2^,^29^. Degradation of the extracellular matrix occurs through cleavage of collagen by collagenases, and progresses via the subsequent degradation of cleaved collagen into gelatin and small peptides by gelatinases^7^,^35^,^36^. Therefore, several studies of wrinkle formation have focused on gelatinases and collagenases^5^,^12^,^13^. Given that MMP-3 is primarily responsible for causing collagen degradation and because MMP-9 plays an important role in the final degradation of fibrillar collagens^4^,^37^, we used collagenase MMP-3 and gelatinase MMP-9 as the major markers of UVB-induced wrinkles.

Here, UVB irradiation induced significant collagenolytic activity and gelatinolytic activity with similar skin localization of MMP-3 and MMP-9. Cilostazol treatment significantly reduced the proteolytic activity of each of the localized MMPs in the dermis. Our results suggest that treatment with cilostazol ameliorates proteolytic activity and subsequently alleviates the degradation of the extracellular matrix. To our knowledge, there is only one published *in vitro* study of the effects of cilostazol on the expression of MMPs in skin cells, showing that cilostazol regulates UVB-induced MMP-1 expression and Type I procollagen synthesis in human dermal fibroblasts^38^,^39^. Cilostazol suppresses the degradation of collagen in human chondrocytes^40^ and reduces MMP-9 promoter activity that contributes to invasiveness in a human monocytic leukemia cell line^41^. Thus, our work provides new *in vivo* evidence for the effect of cilostazol on attenuating UVB-induced proteolytic activity.

Next, we attempted to detect the ROS-related cellular signaling pathway involved in UVB-induced wrinkle formation. UV radiation-induced ROS generation amplifies the cellular signal leading to the expression of MMPs via the MAPK and PI3K/Akt signaling pathways^4^,^7^,^8^. Regulatory sites in the genes encoding MMPs contain an AP-1 regulatory element. Stimulated AP-1 transcription factors, such as c-Jun and c-Fos, bind to target gene promoters, including MMPs, and activate their synthesis^6^,^7^,^42^. Two representative transcription factor families, NF-κB and AP-1, transcribe the genes encoding MMPs^5^,^10^,^11^. Signaling pathways and their interactions with NF-κB and the dual inhibition of AP-1 seem to offer promising strategies for anti-wrinkle treatments^4^.

The levels of UVB-induced phosphorylated p38 were significantly reduced by cilostazol treatment while those of phosphorylated Akt were enhanced. Phosphorylated NF-κB, but not c-Jun and c-Fos, was also downregulated by cilostazol treatment in UVB-irradiated mice. Considering its cellular expression sites, the marked expression of active Akt due to cilostazol treatment may involve protective effects on the epidermis, such as organization of the stratum corneum that regulates trans-epidermal water loss^43^. Further, the marked expression of active p38 MAPK and NF-κB in the dermal layer suggests that reduced phosphorylation of these molecules by cilostazol treatment may be important mediators for controlling expression of MMPs in the dermis, subsequently decreasing the formation of deep wrinkles. In dermal fibroblasts, cilostazol inhibits UVB irradiation-induced phosphorylation of JNK and p38 MAPK as well as that of AP-1^38^. Cilostazol also suppressed the translocation of NF-κB in a human monocytic leukemia cell line^41^. However, western blot and immunohistochemical analyses suggest that the protective effects of cilostazol against wrinkle formation mainly involve p38 MAPK and NF-κB signaling.

The mechanisms underlying the effects of cilostazol on the regulation of ROS by cAMP and MMP signaling in the skin require further investigation. However, in the present study, cilostazol potently inhibited UVB-induced wrinkle formation by inhibiting ROS, p38 MAPK, and NF-κB expression. Our observations indicate that p38 and NF-κB are novel molecular targets of cilostazol for the inhibition of UVB-induced signal transduction, leading to down-regulation of MMPs. Considering that a ROS imbalance correlates with reduced cAMP-dependent phosphorylation in human skin fibroblasts^31^, our results provide some basis for the activation of cAMP-dependent intracellular signaling or of cAMP analogs against UV-induced photoaging. We therefore conclude that cilostazol may protect against wrinkles and other skin damage due to photoaging.

## Methods

### Animals

Female, 8-week-old HR-1 hairless mice were obtained from Dooyeol Biotech (Seoul, Korea), and were acclimated for 1 week before the start of the experiments. The mice were housed at 22 °C under a 12 h dark/light cycle, and were fed a commercial diet and allowed access to tap water *ad libitum* throughout the study. The study was approved by the Institutional Animal Care and Use Committee (IACUC) of Pusan National University (approval number PNU 2015-0847). All animal experiments were conducted in accordance with the IACUC guidelines. The mice were randomly divided into 5 treatment groups (n = 7 mice/group): control, 0.3% cilostazol alone (Cilo0.3), UVB-irradiated alone (UVB), UVB-irradiated plus 0.3% cilostazol (UVB+Cilo0.3), and UVB-irradiated plus 0.5% cilostazol (UVB+Cilo0.5).

### UVB irradiation

UVB irradiation of mice was performed using a UV lamp (Philips, Somerset, NJ, USA) with an emission spectrum between 290 nm and 315 nm. A UV meter (Lutron UV-340A, Taipei, Taiwan) was used for measurement of UV irradiance. Mice were anesthetized by intraperitoneal (i.p.) injection of 8% chloral hydrate (Sigma-Aldrich, St. Louis, MO, USA). The UV lamp was situated 30 cm above the dorsal skin of the mice. UV light was applied to the mice for 8 weeks, and the amount of irradiance was gradually increased from 1 MED up to 4 MED (1 MED = 1 minimal erythema dose = 100 mJ/cm^2^) with no injury. The dorsal skin of the mice was exposed to UV light 3 times per week at 1 MED for the first 2 weeks, 2 MED 3 times per week for 2 weeks, 3 MED 2 times per week for 2 weeks, and 4 MED 2 times per week for 2 weeks.

### Cilostazol treatment

Cilostazol [OPC-13013, 6-[4-(1-cyclohexyl-1H-tetrazol-5-yl) butoxy]-3,4-dihydro-2-(1H)-quinolinone] was donated by Otsuka Pharmaceutical (Tokushima, Japan). Two concentrations (0.3 g and 0.5 g) of cilostazol were prepared by dissolution in a solution of 75 mL ethanol, 1.5 g glycerol, and 10 g propylene carbonate, diluted to 100 mL with distilled water. Dissolved cilostazol was applied on the dorsal skin, which was impregnated with the cilostazol solution twice daily for 8 weeks during UVB irradiation. We also applied dissolved cilostazol only on one side of the dorsal skin during UVB irradiation for 2 weeks to normalize the background signal intensity between the control and test samples obtained from the mice. We exposed the skin to UV light 7 times at 1 MED during the first week and 3 times at 2 MED during the second week.

### Wrinkle measurement

Skin condition was assessed by photographing the mouse dorsal skin at the end of every week to confirm the extent of wrinkle formation. Replicas were prepared using a SILFLO kit (CuDerm Corporation, Dallas, TX, USA) to measure the area and depth of the wrinkles just before the mice were sacrificed. Skin silicon impressions were analyzed by a Visioline VL650 (Courage & Khazaka, Cologne, Germany).

### Histological analysis and collagen staining

The dorsal skin of the mice between the ilium was harvested under anesthesia at the end of the experiment, at an age of 17 weeks. Skins were immersed and fixed in 4% paraformaldehyde and 20-μm cryosections were prepared. Hematoxylin staining (Sigma-Aldrich) was performed to examine histological features and epidermal and dermal thickness. Masson’s trichrome staining was performed using Masson’s trichrome staining kit (Abcam, Cambridge, MA, USA) to examine the density of collagen fibers. After mounting in mounting medium (Vector Laboratories, Inc., Burlingame, CA, USA), images were captured using an Axio Imager A1 microscope (Carl Zeiss Inc., Göttingen, Germany). IMT i-solution (IMT i-solution Inc., Vancouver, BC, Canada) was used for automatic measurement analysis of each staining.

### Assessment of ROS

A ROS assay kit (OxiSelect^TM^ *In vitro* ROS/RNS Assay Kit, Cell Biolabs Inc., San Diego, CA, USA) was used to detect ROS. Briefly, the dorsal skin tissue was rapidly frozen using 2-methylbutane and liquid nitrogen. Tissues were homogenized and centrifuged at 10,000 × *g* for 5 min. Supernatants were removed and stored at −80 °C. In order to quantify both free radicals and hydrogen peroxide (H_2_O_2_), separate standard curves for free radicals and hydrogen peroxide were prepared according to the ROS assay kit manual. A standard curve for 2′, 7′-dichlorodihydrofluorescein (DCF) and one for the detection of H_2_O_2_ was prepared from a DCF standard (0, 1, 10, 100, 1000, and 10’000 nM DCF) and a 2 mM H_2_O_2_ standard (0, 0.039, 0.078, 0.158, 0.313, 0.625, 1.25, 2.5, 5, 10, and 20 μM H_2_O_2_), respectively. The skin sample supernatant and H_2_O_2_ standard were added to 96-well plates. The catalyst (50 μL of a 1- × dilution) was added and was incubated for 5 min under shaking. A DCFH stock solution (100 μL) was added to each well and was reacted for 30 min in the dark. Fluorescence was read on a fluorescence plate reader (Multilabel counter, VICTOR^3^_TM_, Waltham, MA, USA).

For ROS staining, the dorsal skin was rapidly frozen using 2-methylbutane and liquid nitrogen. Cryosections were prepared at a thickness of 20 μm. Sections were incubated with dihydroethidium (DHE; Sigma-Aldrich) in antibody dilution buffer for 2 h at room temperature in the dark. The sections were subsequently washed with phosphate-buffered saline (PBS) and were incubated with DAPI (Vector Laboratories Inc.) in PBS for 10 min. The slides were mounted using Fluorescence-Mounting Medium (Dako, Glostrup, Denmark). An optical microscope (Axio Vision LSM 510, Carl Zeiss Inc.) was used for visualizing fluorescence intensity.

### *In situ* zymography

To detect the activity and location of gelatinase and collagenase, *in situ* zymography was performed. Dorsal skin tissues were rapidly frozen using 2-methylbutane and liquid nitrogen, and 20-μm cryosections were prepared. Sections were reacted at 37 °C for approximately 8 h in dark humidified chambers with a Molecular Probes EnzChek Gelatinase/Collagenase Assay Kit (Life Technologies, Eugene, OR, USA). After washing, the skins were fixed in 10% neutral-buffered formalin in the dark. To evaluate MMP-9 and MMP-3 co-localization, sections were incubated with primary anti-MMP-9 (Millipore, Billerica, MA, USA) or anti-MMP-3 (Santa Cruz Biotechnology Inc., CA, USA) in antibody dilution buffer (1 × PBS, 1% bovine serum albumin [BSA], and 0.3% Triton X-100) overnight in the dark, followed by washing with PBS. The sections were incubated with the corresponding secondary goat anti-rabbit IgG-TR (Vector Laboratories Inc.) or rabbit anti-goat IgG-TR (Vector Laboratories Inc.) antibody for 2 h at room temperature in the dark, followed by washing with PBS. Subsequently, the slides were mounted using mounting medium (Vector Laboratories Inc.), and images were acquired using a fluorescence microscope (Carl Zeiss Imager M1, Carl Zeiss Inc.). IMT i-Solution (IMT i-Solution Inc.) was used for automatic measurement of the integrated optical density (IOD).

### Western blotting

The dorsal skin was homogenized in lysis buffer (250 mM NaCl, 5 mM EDTA, 25 mM Tris-HCl, 1% NP-40, 1 mM PMSF, 5 mM DTT, 0.1 mM Na_3_VO_4_, 10 mM NaF, leupeptin, and protease inhibitor). Equal amounts of total proteins were separated by sodium dodecyl sulfate polyacrylamide gel electrophoresis on a 10–12% gel and were transferred to a nitrocellulose membrane (Whatman, Dassel, Germany). The membranes were blocked with 5% non-fat milk in PBS containing 0.4% Tween-20 (PBS-T). The membranes were subsequently incubated with the following primary antibodies: extracellular signal regulated kinases (ERK; Cell Signaling Technology, Danvers, MA, USA), phospho-ERK (pERK; Cell Signaling Technology), c-Jun N-terminal kinases (JNK; Cell Signaling Technology), phospho-JNK (pJNK; Cell Signaling Technology), p38 (Santa Cruz Biotechnology Inc.), phospho-p38 (pp38; Cell Signaling Technology), Akt (Cell Signaling Technology), phospho-Akt (pAkt; Cell Signaling Technology), PI3K (Cell Signaling Technology), phospho-PI3K (pPI3K; Cell Signaling Technology), c-Jun (Cell Signaling Technology), phospho-c-Jun (p-c-Jun; Cell Signaling Technology), c-Fos (Santa Cruz Biotechnology, Inc.), NF-κB (Abcam), phospho-NF-κB (pNF-κB; Cell Signaling Technology), or MMP-9 (Millipore) at 4 °C overnight in 5% non-fat milk in PBS-T. Subsequently, the membranes were incubated with the corresponding secondary antibody. Secondary antibodies were purchased from Santa Cruz Biotechnology. Beta-actin (Sigma-Aldrich) was used as a loading control for all experiments. Quantification of immunoreactivity was performed by densitometric analysis of the protein bands using an Image Quant LAS 4000 (Fujifilm, Tokyo, Japan).

### Immunohistochemistry

The skins were fixed in 4% paraformaldehyde and 20-μm cryosections were prepared. The sections were incubated with a blocking buffer (1 × PBS/5% normal goat serum/0.3% triton X-100) for 1 h. The sections were incubated with the following primary antibodies at 4 °C overnight in PBS: pAkt (Cell Signaling Technology), pp38 (Cell Signaling Technology), and pNF-κB (Cell Signaling Technology). After washing with PBS, the sections were incubated with fluorescent secondary antibody (Vector Laboratories Inc.) for 2 h in the dark. The slides were mounted with mounting medium (Vector Laboratories Inc.). An optical microscope (Axio Vision LSM 510, Carl Zeiss Inc.) was used for measuring the fluorescence intensity.

### Statistical analysis

The data are expressed as the mean ± SEM. The SigmaStat statistical program (version 11.2; Systat Software, San Jose, CA, USA) was used for statistical analysis of the data. All statistical analyses were performed using one-way ANOVA with Tukey’s post-hoc test. A value of *P* < 0.05 was considered statistically significant.

## Additional Information

**How to cite this article**: Kim, H. N. *et al*. Anti-photoaging properties of the phosphodiesterase 3 inhibitor cilostazol in ultraviolet B-irradiated hairless mice. *Sci. Rep.* **6**, 31169; doi: 10.1038/srep31169 (2016).

## Supplementary Material

Supplementary Information
