# Supplementary material for: Anti-photoaging properties of the phosphodiesterase 3 inhibitor cilostazol in ultraviolet B-irradiated hairless mice
Source: Sci Rep. 2016 Aug 3;6:31169. doi: 10.1038/srep31169 (PMC4971469; doi:10.1038/srep31169)
Supplement: Supplementary Information [file srep31169-s1.pdf]

Supplementary Information

Anti-photoaging properties of the phosphodiesterase 3 inhibitor cilostazol in ultraviolet B-irradiated hairless mice

Ha Neui Kim[1,2], Chan Hee Gil[1], Yu Ri Kim[1,2], Hwa Kyoung Shin[1,2,3], Byung Tae Choi[1,2,3,*]

[1]Department of Korean Medicine, School of Korean Medicine, Pusan National University, Yangsan 50612, Korea

[2]Korean Medical Science Research Center for Healthy-Aging, Pusan National University, Yangsan 50612, Korea

[3]Division of Meridian and Structural Medicine, School of Korean Medicine, Pusan National University, Yangsan 50612, Korea

[*] Corresponding author: Dr. Byung Tae Choi

Department of Korean Medical Science, School of Korean Medicine, Pusan National University, Yangsan 50612, Republic of Korea

Telephone: +82-51-510-8475; Fax: +82-51-510-8437; E-mail: choibt@pusan.ac.kr

Supplementary Information Figure S1

A

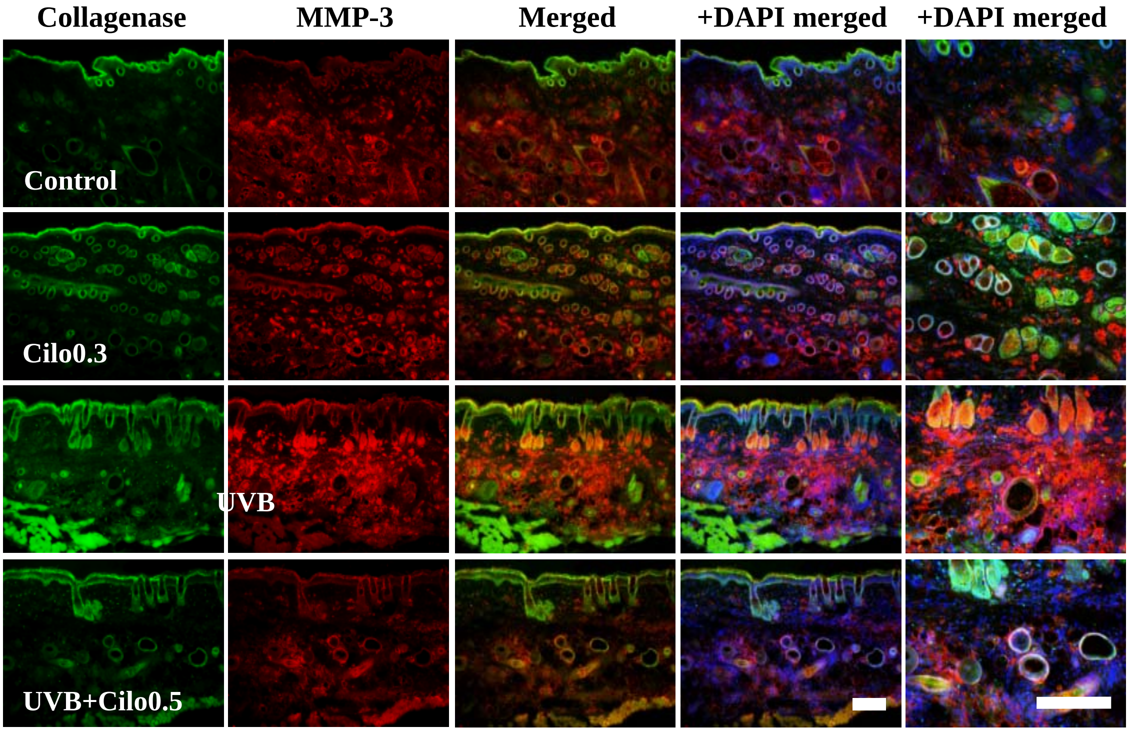

B

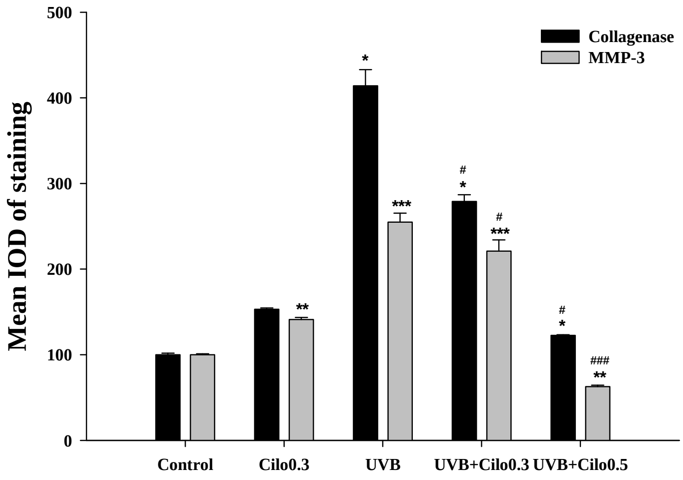

Supplementary Figure 1. Effect of cilostazol on collagenase and MMP-3 activity in the dorsal skin of UVB-irradiated mice. (A) Images of *in situ* zymographic analysis for collagenase and MMP-3 and (B) histogram analysis of IOD measurement. Treatment with cilostazol significantly suppressed collagenase activity and MMP-3 expression in the dorsal skin of UVB-irradiated mice. $^{*}P$<0.05, $^{**}P$<0.01, and $^{***}P$<0.001 vs. control mice; $^{\#}P$<0.05 and $^{\#\#\#}P$<0.001 vs. UVB-irradiated mice. Scale bar = 200 μm.
